# Homocysteine and Familial Longevity: The Leiden Longevity Study

**DOI:** 10.1371/journal.pone.0017543

**Published:** 2011-03-08

**Authors:** Carolien A. Wijsman, Diana van Heemst, Maarten P. Rozing, P. Eline Slagboom, Marian Beekman, Anton J. M. de Craen, Andrea B. Maier, Rudi G. J. Westendorp, Henk J. Blom, Simon P. Mooijaart

**Affiliations:** 1 Department of Gerontology and Geriatrics, Leiden University Medical Center, Leiden, The Netherlands; 2 Section of Molecular Epidemiology, Department of Medical Statistics, Leiden University Medical Center, Leiden, The Netherlands; 3 Netherlands Consortium of Healthy Aging (NCHA), Leiden, The Netherlands; 4 Metabolic Unit, Department of Clinical Chemistry, Institute for Cardiovascular Research, VU Medical Center, Amsterdam, The Netherlands; Erasmus University Medical Center, Netherlands

## Abstract

Homocysteine concentrations are a read-out of methionine metabolism and have been related to changes in lifespan in animal models. In humans, high homocysteine concentrations are an important predictor of age related disease. We aimed to explore the association of homocysteine with familial longevity by testing whether homocysteine is lower in individuals that are genetically enriched for longevity. We measured concentrations of total homocysteine in 1907 subjects from the Leiden Longevity Study consisting of 1309 offspring of nonagenarian siblings, who are enriched with familial factors promoting longevity, and 598 partners thereof as population controls. We found that homocysteine was related to age, creatinine, folate, vitamin B levels and medical history of hypertension and stroke in both groups (all p<0.001). However, levels of homocysteine did not differ between offspring enriched for longevity and their partners, and no differences in the age-related rise in homocysteine levels were found between groups (p for interaction 0.63). The results suggest that homocysteine metabolism is not likely to predict familial longevity.

## Introduction

Healthy longevity is a complex phenotype determined by a mix of genetic and environmental factors. The heritable component of human longevity is estimated to be modest (25–30%) [1], [2]. This genetic advantage in lifespan is not explained by common disease alleles [3], suggesting the existence of specific longevity promoting mechanisms. In humans, little is known about the mechanisms driving familial longevity. Data from various model organisms suggest that longevity is promoted by alterations in insulin signaling, metabolism and increased resistance to oxidative stress. Recent studies have demonstrated that growth hormone, thyroid hormone and insulin may play an important role in the control of the key proteins that regulate maintenance of homocysteine homeostasis. For example, Ames Dwarf mice, which lack the pituitary hormones growth hormone, prolactin, and thyroid stimulating hormone and show extended lifespan compared to their wild-type counterparts, were found to exhibit upregulated transsulfuration and low homocysteine levels, which might be linked to enhanced cellular oxidative stress defense mechanisms [4], [5].

In humans, data regarding methionine metabolism and longevity are sparse. Increased concentrations of homocysteine have consistently been associated with ischemic cardiac events, stroke, venous thrombosis, Alzheimer's disease, osteoporosis and depression [6]–[11]. In line with these associations, higher levels of homocysteine have shown to independently predict all cause mortality in large population cohorts [10], [12], [13] as well as in clinical populations [14], [15]. Particularly, in the oldest old homocysteine has shown to be a better predictor for all cause and cardiovascular mortality than the commonly used Framingham risk score [16]. While the association between homocysteine and disease is well established in the general population, it is unclear whether familial longevity is also characterised by altered homocysteine levels.

We investigated whether lower levels of homocysteine at middle age mark familial longevity. In the Leiden Longevity Study, we have previously shown that the offspring from long-lived nonagenarian sibling have, at middle age, a lower mortality rate compared to the normal population, and have a healthier metabolic profile and lower prevalence of cardiovascular disease compared to their partners with whom they share their environment [17], [18].

## Materials and Methods

### Study design and population

The Leiden Longevity Study comprises 421 families, as described more extensively elsewhere [17]. Families were included and regarded as enriched for familial longevity if at least two long lived siblings were alive and fulfilled the age-criterion of 89 years or older for males and 91 year or older for females. As no proper controls exist for this age group, for further studies the offspring of these long-lived nonagenarians were included with their partners as controls. This generation carries on average 50% of the genetic propensity of their long lived parent and were shown to have a lower mortality (SMR 0.65) compared with their birth cohort [17]. Their partners, with whom most had had a relationship for decades, were included as environmentally matched controls, thereby minimizing environmental differences between the groups under study. We have previously shown that environmental factors between groups, such as smoking and physical activity, did not differ between groups [19]. There were no selection criteria on health or demographic characteristics. In total, 1671 offspring and 744 controls were included in the Leiden Longevity Study. For the present study, we determined homocysteine concentrations in 1907 subjects: 1309 offspring of long-lived siblings and 598 partners thereof, of whom data on comorbidity and medication use were available. The Medical Ethical Committee of the Leiden University Medical Center approved the study and written informed consent was obtained from all participants.

### Blood samples and homocysteine measurement

At baseline, all participants visited the study center. Non-fasted plasma samples were taken for the determination of total homocysteine concentration. Total homocysteine was measured using a competitive immunoassay (Architect, Abbott Laboratories, Illinois USA). Creatinine was measured by Kinetic Alkaline Picrate methodology. Folate and Vitamine B12 were measured with respectively the Abbott Architect Folate and Abbott Architect B12 assay (Abbott, Abbott Park, USA), which use the Chemiluminescent Microparticle Immunoassay (CMIA) technology with flexible assay protocols, referred to as Chemiflex. Creatine, vitamine B12 and folate measurements were implemented on an Abbott ci8200.

### *MTHFR* Genotype

Genotyping of the rs1801133 in the *MTHFR* gene, encoding the enzyme methyl-tetrahydrofolate-reductase, commonly known as MTHFR C677T, was performed using Sequenom MassARRAY iPLEX®Gold. The high iPLEX primer design was performed by entering the sequences encompassing each polymorphism into SpectroDESIGNER provided by Sequenom®, Inc. (CA, USA). The high plex reaction protocol was used (www.sequenom.com/iplex). The average genotype call rate for genotyped SNPs was 96.3% and the average concordance rate was 99.7% among 4% duplicated control samples.

### Comorbidity and medication use

Additional information was collected from the generation of offspring and partners, including self-reported information on lifestyle factors such as smoking. Information on medical history was obtained from the participants' treating physicians, including history of myocardial infarction, stroke, hypertension, diabetes, malignancy, rheumatoid arthritis and COPD. Information on medication use was obtained from the participants' pharmacists.

### Statistical analysis

Homocysteine concentrations were normally distributed. If not otherwise stated, data are presented as mean with standard error of the mean (S.E.). For multivariate analyses, we used ANOVA to calculate differences between groups. For analyses of differences between the offspring and controls, we calculated robust standard errors to adjust for family relationship. A P-value<0.05 was regarded statistically significant. All analyses were performed using SPSS version 17.0. Robust standard errors were calculated using Stata version 10.

## Results

Baseline characteristics of participants are shown in Table 1. At baseline, offspring were slightly older, had a lower prevalence of hypertension and diabetes mellitus and a tendency towards lower prevalence of myocardial infarction compared to controls. Creatinine levels tended to be lower in offspring (p = 0.076), whereas folic acid and vitamine B12 did not differ between groups. The common C677T polymorphism in the *MTHFR* gene was equally distributed between groups. Allele frequency in our cohort was comparable to earlier reports in the Dutch population [20] and genotype distribution was in Hardy Weinberg equilibrium.

**Table 1:** Baseline characteristics.

Characteristics	Offspring	Controls	P-value
	n = 1306	n = 598	
Female sex, n (%)	701 (53.6)	342 (57.2)	0.13
Age, mean (SD)	59.3 (6.5)	58.6 (7.2)	0.076
History of, n (%)			
Myocardial infarction	30 (2.3)	23 (3.8)	0.055
Stroke	44 (3.4)	16 (2.7)	0.42
Hypertension	296 (22.6)	161 (26.9)	**0.038**
Diabetes Mellitus	57 (4.4)	45 (7.5)	**0.004**
Malignancy	112 (8.6)	43 (7.2)	0.32
COPD	45 (3.4)	24 (4.0)	0.55
Reumatoid Arthritis	18 (1.4)	4 (0.7)	0.18
Current smoking	143 (10.9)	75 (12.5)	0.31
Creatinine (mmol/L)	81.7 (12.6)	82.1 (13.6)	0.076
Folate (nmol/L)	10.4 (6.0)	10.5 (6.3)	0.66
Vitamin B12 (pmol/L)	369.0 (141.9)	358.8 (157.6)	0.13
MTHFR C677T genotype, n (%)			
677CC	537 (41.1)	245 (41.0)	0.70
677CT	570 (43.6)	278 (46.5)	
677 TT	155 (11.9)	67 (11.2)	

Data are presented as means with standard deviation or number and percentage when appropriate. P values represent difference between groups as measured by ANOVA with correction for age and sex when appropriate, or chi square for proportion. COPD = chronic obstructive pulmonary disease.

First, we tested whether homocysteine concentrations were related to well known determinants. Mean homocysteine concentration was 12.5 µmol/L (standard deviation (SD) 2.9 µmol/L). Mean homocysteine concentrations were 11.8 µmol/L (standard error (SE) 0.09 µmol/L) for females, and 13.3 µmol/L (SE 0.09 µmol/L) for males (p-value for difference<0.001). Homocysteine concentrations also related to calendar age, with a mean increase of 0.06 µmol/L (SE 0.01 µmol/L) per year for women (p<0.001), and 0.07 µmol/L (S.E.0.01) per year increase in tHcy for men (p<0.001). Furthermore, homocysteine concentrations were positively correlated with creatinine levels (p<0.001), folic acid (p<0.001), vitamin B12 levels (p<0.001), and genotype of the *MTHFR* C677T polymorphism (p<0.001).

We then tested whether homocysteine concentrations were related to prevalence of disease history. Table 2 shows that homocysteine concentrations were significantly higher when history of stroke or hypertension was present.

**Table 2:** Association between homocysteine concentration and prevalence of disease history.

History of disease	n	Homocysteine (mean, SE in umol/mol)	p-value
Myocardial infarction			
absent	1841	12.7 (0.08)	0.14
present	53	14.1 (0.5)	
Stroke			
absent	1838	12.6 (0.08)	**0.009**
present	60	14.3 (0.5)	
Hypertension			
absent	1408	12.5 (0.09)	**0.009**
present	456	13.2 (0.2)	
Diabetes Mellitus			
absent	1770	12.7 (0.08)	0.19
present	102	13.4 (0.4)	
Malignancy			
absent	1732	12.7 (0.08)	0.62
present	155	13.0 (0.3)	
COPD			
absent	1812	12.7 (0.08)	0.11
present	69	13.6 (0.4)	
Reumatoid Arthritis			
absent	1862	12.7 (0.08)	0.26
present	22	12.0 (0.8)	

Data are presented as mean with standard error. P-values obtained after linear regression with adjustment for age and sex.

**Figure 1:**
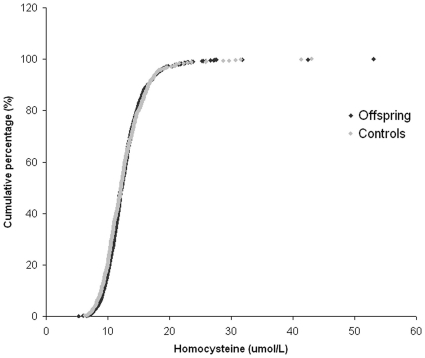
Cumulative distribution curve for homocysteine in offspring (n = 1306) and controls (n = 598). Each dot represents one individual.

Finally, we tested whether differences existed in homocysteine concentrations between offspring and controls. Table 3 shows the differences between offspring and partner, both crude and after adjustment for age, sex and creatinine. No differences were found in homocysteine concentrations between offspring and partners (12.7 (SE 0.1) vs. 12.7 (SE 0.1) µmol/L). Furthermore, no differences in distribution of homocysteine were found between groups, showing the lack of difference of homocysteine between groups across the entire range of values (Figure 1). Also, no differences were found when stratifying for males and females separately. Additionally, no differences were found in the age-related rise in homocysteine between offspring and controls (p for interaction 0.63). In the total study population, 21 subjects had prescriptions for folic acid or vitamin B12 supplementation. Excluding these subjects from analysis did not influence outcomes. To assess the effect of outlying high homocysteine values, we repeated the analysis excluding 18 subjects with homocysteine concentrations higher than the 99^th^ percentile (25.78 µmol/L), which did not materially change results (data not shown).

**Table 3:** Homocysteine levels in offspring and controls.

	Homocysteine (µmol/L)		
	offspring	controls	p-value
All			
Crude	12.7 (0.1)	12.7 (0.1)	0.92
Adjusted	12.7 (0.1)	12.7 (0.1)	0.78
Males			
Crude	13.4 (0.1)	13.8 (0.2)	0.13
Adjusted	13.4 (0.1)	13.6 (0.1)	0.41
Females			
Crude	12.1 (0.1)	11.9 (0.2)	0.42
Adjusted	12.1 (0.1)	12.0 (0.2)	0.71

Data represent adjusted means with standard error. P-value obtained after correction for family clusters within offspring. Adjusted data obtained after correction for sex, age and creatinine.

## Discussion

In the present study we aimed to find a relationship between familial longevity and homocysteine concentrations. We found that homocysteine was associated with classical determinants, but did not differ between individuals from long-lived families compared to their environmentally matched partners, nor did age-related increase in homocysteine concentrations.

We found a positive association between plasma homocysteine concentrations with cardiovascular disease prevalence, and with classical determinants of homocysteine such as age, sex, creatinine, vitamin B12 and folate, and *MTHFR* genotype. These findings implicate that homocysteine measurements were valid and show associations that are in agreement with previous reports [10], [21]–[23].

Unlike earlier observations of difference in glucose, lipid and thyroid metabolism [18], [19], [24], [25], we found no association of levels of homocysteine with familial longevity at middle age in our study population. This finding is in line with the recent debate on the causality of homocysteine in cardiovascular disease. Although homocysteine has consistently shown to predict cardiovascular disease in prospective studies, recent meta-analyses and reviews of the homocysteine-lowering trials showed no effect on cardiovascular end points of homocysteine lowering by means of folate and vitamin B in individuals with prior cardiovascular disease [26], [27]. However, controversy on the implications of these findings for the causal role of homocysteine in cardiovascular disease remain, for instance because potential adverse side effects of folate and B vitamins on atherosclerosis might undo the beneficial effects of lowering homocysteine [28]. It has been proposed that homocysteine is a marker or read-out of vascular damage and that homocysteine *per se* is not causally involved in the etiology of cardiovascular disease and mortality. The data presented in the present study support this theory. Despite the lower prevalence of cardiovascular disease and cardiovascular risk factors in our cohort of individuals from long-lived families, we did not find homocysteine concentrations to be lower in middle-aged subjects enriched for longevity.

Genetic studies of *MTHFR* polymorphisms, a genetic determinant of homocysteine concentrations, have shown a relationship between *MTHFR* genotype and coronary heart disease [29], although substantial heterogeneity between the results of published studies reflected selective publication or other methodological problems. These findings further fuel the debate on the causal role of homocysteine in cardiovascular disease. In the present study we used the association of MTHFR genotypes with homocysteine concentrations in this study as positive control, to show that homocysteine concentrations showed expected associations and is therefore a valid measurement. Notably, we did not perform a formal Mendelian Randomization analysis in our family based approach as we have selected groups based on phenotypic differences, namely longevity. Furthermore, the study was underpowered to perform such an analysis. We do therefore not make a claim on causal relationships (or absence thereof) based on this genetic association. Taken together we conclude that levels of homocysteine do not predict familial longevity.

A drawback of our study is that we did not measure other metabolites of the methionine cycle such as methionine, cysteine and glutathione and did therefore not fully cover the methionine metabolism. Glutathione, the end point of the transsulfuration pathway of homocysteine, could be of interest as it is an important intra-cellular antioxidant and is related to aging and disease in humans and longevity in various animal models [30]–[32]. Other interesting metabolites in the methionine cycle include S-adenosyl methionine (SAM) and S-adenosylhomocysteine (SAH), as their ratio is a representation of methylation status, and both markers have been associated with cardiovascular abnormalities more strongly than homocysteine itself [33]–[35]. For that reason, we cannot exclude that other differences in methionine metabolism might be related to familial longevity. Still, given the lack of difference in homocysteine concentrations, which represent a central marker of methionine metabolism, we consider the possibility of finding major differences in other metabolites to be small.

The Leiden Longevity Study is unique in that it allows studying us to study longevity-related factors already at middle-age. To our knowledge, our study is the first to address homocysteine in relation to familial longevity. In conclusion, the results suggest that homocysteine metabolism is not likely to predict familial longevity.
